# Heritable Differences in Catecholamine Signaling Modulate Susceptibility to Trauma and Response to Methylphenidate Treatment: Relevance for PTSD

**DOI:** 10.3389/fnbeh.2019.00111

**Published:** 2019-05-17

**Authors:** Jessica Deslauriers, Mate Toth, Xianjin Zhou, Victoria B. Risbrough

**Affiliations:** ^1^Department of Psychiatry, University of California, San Diego, San Diego, CA, United States; ^2^Center of Excellence for Stress and Mental Health, Veterans Affairs Hospital, La Jolla, CA, United States; ^3^Department of Behavioural Neurobiology, Institute of Experimental Medicine, Hungarian Academy of Sciences, Budapest, Hungary

**Keywords:** COMT, catecholamine, PTSD, predator stress, methylphenidate

## Abstract

Alterations in cortical catecholamine signaling pathways can modulate acute and enduring responses to trauma. Heritable variation in catecholamine signaling is produced by a common functional polymorphism in the catechol-O-methyltransferase (COMT), with Val carriers exhibiting greater degradation of catecholamines than Met carriers. Furthermore, it has recently been suggested that drugs enhancing cortical catecholamine signaling may be a new therapeutic approach for posttraumatic stress disorder (PTSD) patients. We hypothesized that heritable differences in catecholamine signaling regulate the behavioral response to trauma, and that methylphenidate (MPD), a drug that preferentially blocks catecholamine reuptake in the prefrontal cortex (PFC), exerts COMT-dependent effects on trauma-induced behaviors. We first examined the contribution of the functional mutation COMTval158met to modulate enduring behavioral responses to predator stress in a unique “humanized” COMTval158met mouse line. Animals were exposed to a predator (cat) for 10 min and enduring avoidance behaviors were examined in the open field, light-dark box, and “trauma-reminder” tests 1–2 weeks later. Second, we examined the efficacy of chronic methylphenidate to reverse predator stress effects and if these effects were modulated by COMTval158met genotype. Mice were exposed to predator stress and began treatment with either saline or methylphenidate (3 mg/kg/day) 1 week after stress until the end of the testing [avoidance behaviors, working memory, and social preference (SP)]. In males, predator stress and COMTval158met had an additive effect on enduring anxiety-like behavior, with Val stressed mice showing the strongest avoidance behavior after stress compared to Met carriers. No effect of COMT genotype was observed in females. Therefore methylphenidate effects were investigated only in males. Chronic methylphenidate treatment reversed the stress-induced avoidance behavior and increased social investigation independently of genotype. Methylphenidate effects on working memory, however, were genotype-dependent, decreasing working memory in non-stressed Met carriers, and improving stress-induced working memory deficit in Val carriers. These results suggest that heritable variance in catecholamine signaling modulates the avoidance response to an acute trauma. This work supports recent human findings that methylphenidate might be a therapeutic alternative for PTSD patients and suggests that methylphenidate effects on anxiety (generalized avoidance, social withdrawal) vs. cognitive (working memory) symptoms may be modulated through COMT-independent and dependent mechanisms, respectively.

## Introduction

Posttraumatic stress disorder (PTSD) affects 7%–8% of the American population while rates are as high as 22% in veterans (Kessler et al., 2010). In the US, 50%–90% of the population endorse at least one trauma in their lifetime, but only 10%–40% of individuals who endorsed sufficiently high numbers of traumatic events develop PTSD symptoms (Kessler et al., 2010; Kilpatrick et al., 2013), characterized by intrusive re-experiencing of a trauma event, avoidance of trauma-related cues and hyperarousal (Neuner et al., 2004; Kolassa et al., 2010). Since only a fraction of individuals who experience trauma develop PTSD, a better understanding for the biological risk factors that contribute to susceptibility to stress and PTSD will be crucial for the development of novel preventative or early intervention therapeutics.

Cortical catecholamine signaling is critical for acute and enduring responses to trauma (Arnsten, 2009). In humans, heritable variation in peripheral and central catecholamine signaling is produced by the common carried val158met functional single nucleotide polymorphism (SNP; rs4680) in the catechol-O-methyltransferase (COMT) gene, localized on chromosome 22q11.2. The COMTval158met SNP is a coding variant with known functional effects, with COMT being an enzyme that degrades catecholamines: epinephrine, norepinephrine, and dopamine. In the coding sequence for COMT, a valine (Val) is substituted by a methionine (Met) at amino acid residue 158, resulting in a 40% reduction in COMT enzymatic activity in the prefrontal cortex (PFC) of human Met/Met carriers. Therefore, Val/Val carriers have increased catecholamine clearance and reduced catecholamine tone in the cortex (Chen et al., 2004). Preclinical studies of this SNP confirms that it results in reduced enzymatic activity as well as more rapid degradation of the COMT enzyme (Tunbridge, 2010; Risbrough et al., 2014). COMTval158met SNP, which has been associated with a greater risk of PTSD in some, but not all candidate gene studies (Boscarino et al., 2011; Clark et al., 2013; Almli et al., 2014; Goenjian et al., 2015) is yet to be confirmed in larger genome-wide association studies (GWAS; Nievergelt et al., 2015; Li et al., 2016; Duncan et al., 2018). These discrepancies may be due in part to relatively small effect sizes associated with common SNPs, differences in PTSD outcome measures and differences in the ancestral background across studies (Kolassa et al., 2010; Boscarino et al., 2011; Valente et al., 2011; Arnsten et al., 2015). Contributions of genetic variance in catecholamine signaling to PTSD risk are also likely *via* gene × environment interactions (i.e., timing and intensity of trauma exposure). Animal models of the COMTval158met mutation will be critical in evaluating any causal contributions and mechanisms of heritable differences in catecholamine signaling to enduring stress response because only animal models can enable isolation of genetic effects against a fixed genetic background and “trauma-type” of fixed intensity and time-course.

Only 60% of PTSD patients adequately respond to the existing pharmacological treatments, antidepressants, and approximately 20%–30% of them achieve full remission (Ravindran and Stein, 2010; Steckler and Risbrough, 2012). Thus, there is a major need for novel treatment strategies (Krystal et al., 2017). Evidence suggests that cortical dopamine-enhancing treatments may be a novel therapeutic alternative for PTSD. The psychostimulant methylphenidate (MPD; Ritalin), a dopamine and norepinephrine transporter inhibitor, is a first-line therapeutic for attention deficit hyperactivity disorder (ADHD). Methylphenidate preferentially increases dopamine signaling in the frontal cortex over striatal release when used at low-doses (Koda et al., 2010; Swanson et al., 2011). Interestingly, methylphenidate reverses the enduring anxiety-like traits in animal models of PTSD (Aga-Mizrachi et al., 2014; Ritov and Richter-Levin, 2017), and a small randomized placebo-controlled trial has also reported decreased PTSD symptoms following methylphenidate treatment (McAllister et al., 2016). These effects are yet to be confirmed in larger randomized controlled trials, and the effects of cortical dopamine-enhancing drugs remain largely unexplored in PTSD patients. More importantly, it is unknown how heritable variance in COMT function modulates treatment response to methylphenidate.

To address the role of the heritable variation in COMT function in response to trauma and methylphenidate treatment, we used a “humanized” *COMT*val158met mouse line, in which either the human Val or Met version of the COMT gene is knocked into the mouse locus (mouse COMT has 70% homology with the human gene). In agreement with the known 40% decrease of COMT enzymatic activity in PFC (Chen et al., 2004) and cognitive traits in human Met/Met carriers (Diaz-Asper et al., 2008; Quednow et al., 2009), Met/Met mice display a 30% reduction in enzymatic activity, and exhibit similar cognitive phenotypes (i.e., higher working memory and fear conditioning compared to Val/Val carriers; Risbrough et al., 2014). These behavioral and neurobiological similarities suggest this “humanized” mouse line as a predictive model of heritable differences in COMT function found in humans and thus offer rigorous causal testing of the role of this variation in susceptibility to trauma events and in treatment response. We hypothesized that heritable variance in COMT function regulates the response to a severe trauma by increasing susceptibility to the enduring effects of trauma. We also hypothesized that COMTval158met may modulate the response to methylphenidate treatment. To test these hypotheses we used a predator stress model of PTSD to examine the sensitivity of mice “humanized” for the *COMT*val158met polymorphism to a traumatic event in adulthood (Adamec et al., 2009; Bakshi et al., 2012; Deslauriers et al., 2018). We utilized two different stress protocols, full and protected predator exposure, to examine how COMTval158met modulates response to both high and low “trauma” conditions. We also examined the efficacy of chronic low-dose treatment with methylphenidate on trauma-induced effects on several PTSD-relevant domains, mainly generalized avoidance, social withdrawal and deficits in working memory. Finally, we tested the hypothesis that COMTval158met would modulate methylphenidate efficacy across anxiety (generalized avoidance, social withdrawal) and cognitive (working memory) domains.

## Materials and Methods

### Animals

The humanized *COMT* mouse line was generated as previously described (Risbrough et al., 2014). Heterozygous *COMT* Met/Val breeders were used to produce Met/Met and Val/Val littermates with *N* > 9 backcross to a C57Bl6J background. All subjects were group housed (3–4 per cage) after weaning (PND28) in a temperature controlled (21–22°C) room under a reverse 12 h light/dark cycle (lights off at 7:00 AM). One week before stress exposure, mice were single housed, as it has been shown that predator stress had stronger effects in isolated mice due to lower baseline levels of avoidance behaviors (Adamec et al., 2009). Mice were tested after reaching adulthood (90–95 days old at start of testing). The behavioral tests were performed from 9:00 AM to 12:30 PM, except the social preference (SP) test that was run from 9:00 AM to 6:00 PM and conducted in accordance with the *Principles of Laboratory Animal Care*, National Institutes of Health guidelines, as approved by the University of California San Diego Institutional Animal Care and Use Committee (IACUC, Protocol S09179). To reduce the stress related to transport and new environment, mice were habituated to the testing room for 60 min under red light (15 lux) conditions, with *ad libitum* access to food and water, before each behavioral assessment.

### Exploratory Behavior and Locomotor Activity

**Figure 1:**
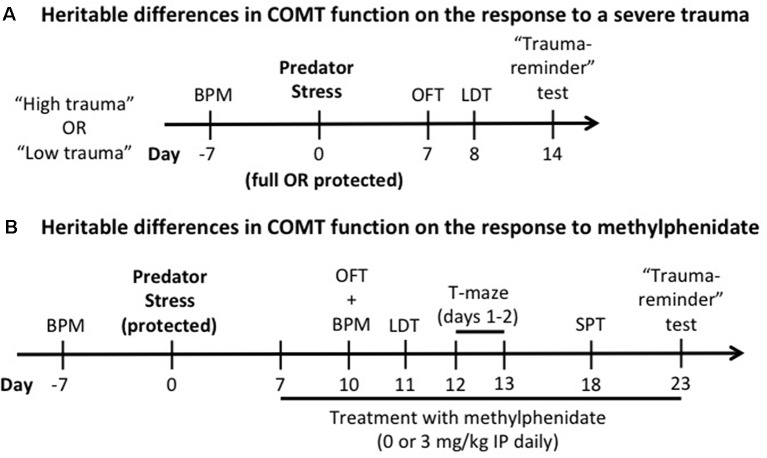
Experimental design. The effects of heritable differences in catechol-O-methyltransferase (COMT) function on the response to a trauma **(A)** and to chronic administration of methylphenidate **(B)** were determined. In both experiments, a week before predator stress exposure, Met/Met and Val/Val carriers were tested in the behavioral pattern monitor (BPM) a week before predator stress exposure to counterbalance all the groups for baseline locomotor activity/exploratory behavior. OFT, open field test; LDT, light-dark box test; IP, intraperitoneal; SPT, social preference test.

Differences in exploratory and locomotor activities are a potential confound for the interpretation of the behavioral outcomes described in the experimental timeline (Figure 1). To counterbalance all the groups for baseline locomotor activity/exploratory behavior, these were assessed in behavioral pattern monitor (BPM) chambers (San Diego Instruments, San Diego, CA, USA; Risbrough et al., 2006) 1 week before stress exposure for all experiments. Each BPM chamber is a clear Plexiglas box containing a 30 × 60 cm holeboard floor. A grid of 12 × 24 photobeams 1 cm above the holeboard floor providing a resolution of 1.25 cm (+16 beams detecting rears) allows us to locate the mouse. Mice were placed in the BPM chambers (under light) and total distance traveled and exploratory behavior (transitions) were recorded for 60 min (Figure 1). In all experiments, there was no effect of genotype and no pre-manipulation group effects of stress or methylphenidate on locomotor activity (counts; Supplementary Figure S1).

### Experiment 1: Effects of Heritable Differences in COMT Function on the Response to Low and High Intensity Trauma

#### Experimental Design and Predator Stress Exposure

**Figure 2:**
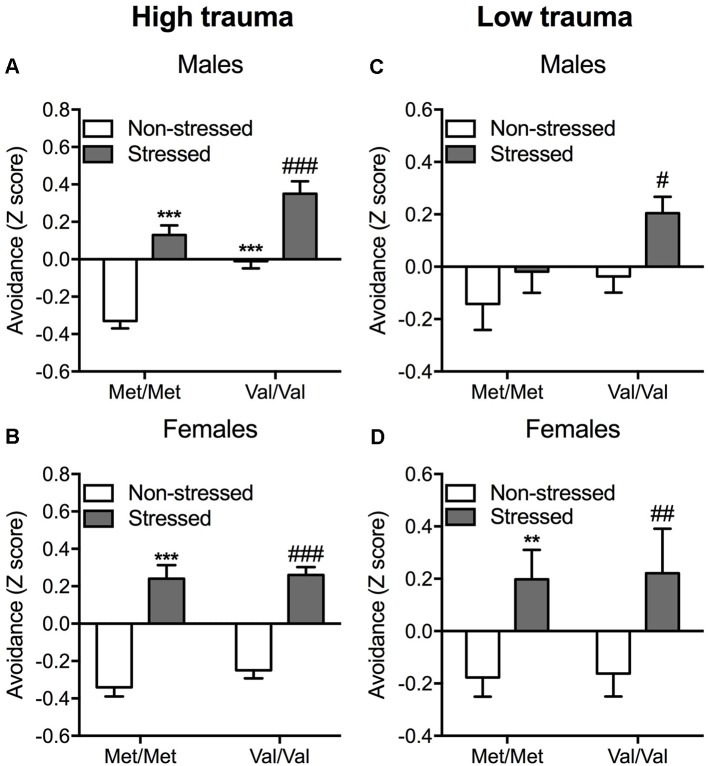
Enhanced avoidance behavior induced by COMT Val/Val genotype and exposure to a predator in mice. A composite *z*-score combining all the parameters across the open field, the light-dark box and the “trauma-reminder” (7, 8, and 14 days post stress respectively) testing paradigms was calculated. Data are presented as mean ± standard error of the mean (SEM) for full (high) trauma **(A,B)** and protected (low) trauma **(C,D)** in both male and female mice. **p < 0.01 and ***p < 0.001 vs. non-stressed Met/Met mice; ^#^*p* < 0.05, ^##^*p* < 0.01 and ^###^*p* < 0.001 vs. non-stressed Val/Val group (*n* = 8–14 per group).

The predator stress paradigm is known to induce several behavioral and biological phenotypes relevant to PTSD (for review, see Deslauriers et al., 2018). The predator stress exposure was performed from 9:00 AM to 6:00 PM as previously described (Toth et al., 2016) using two alternate protocols to manipulate trauma severity. In the full exposure condition (“high trauma”) the mouse was individually placed with a cat (Liberty Research, Waverly, NY, USA) in a well-lit room (2.3 × 1.8 m; 150–200 lux) for 10 min in which both were allowed to freely explore and interact. Predator engagement was measured as described previously (Adamec et al., 2006b; Toth et al., 2016; Deslauriers et al., 2017): sniffing (bringing its nose near the mouse to explore the mouse’s scent), pawing (playing gently with the mouse using its paws), and mouthing (touches the mouse with its mouth without biting). In the protected exposure condition (“low trauma”), the mouse was individually placed in a transparent cage with a wire lid, allowing the cat to freely move around and interact with the cage without being able to touch the mouse directly. This modified protocol aimed to avoid ceiling effects of predator stress as previously described (Adamec et al., 2006a). We based the protected vs. unprotected comparison on (Adamec et al., 2006a, b), which showed that the “low intensity” protected exposure enabled detection of mechanisms of “risk” using 5-HT transporter mutant mice. Both protocols show significant effects of stress (indicating predator exposure was adequate to induce long-term effects), however, the shift in avoidance was stronger in the unprotected version (0.3 vs. 0.6 standard deviation shift between non-stressed and stressed groups, see Figure 2). Thus, the two protocols differ in strength of trauma exposure to induce PTSD-like phenotype as operationalized in this model with avoidance behavior from 7 to 14 days after exposure. Only one cat is used per exposure, and the cat is exposed to 3–6 mice per day and there is an 1-h break between exposures to prevent habituation. COMT Met/Met and Val/Val mice were assigned to non-stressed or stressed groups (*n* = 85, 8–14 per group per sex for full exposure; *n* = 99, 10–18 per group per sex for protected exposure). Non-stressed mice were handled for 1 min. Generalized avoidance behaviors were assessed as described in Figure 1A.

#### Open Field Test

Open field test (PFT) was performed in an open arena (40 × 40 × 40 cm; 800 lux). Mice were placed in a corner and their activity was assessed for 10 min. Total distance traveled, entries into and time exploring the center area (25 × 25 cm), and latency of the first entry in the center area were analyzed using Ethovision Tracking Software (Noldus, Leesburg, VA, USA).

#### Light-Dark Box Test

The open field arena was divided in two 20 × 40 × 20 cm chambers (one well-lit; 950 lux and one covered; <5 lux) joined by a 6 × 6 cm door. Mice were placed in the dark chamber with door closed for 30 s and the test was started by opening the door. For 10 min, the number of entries into and time spent in the light chamber, and the latency of the first entry were assessed using Ethovision Tracking Software.

#### “Trauma-Reminder” Test

To measure avoidance of trauma-associated cues (Toth et al., 2016; Deslauriers et al., 2017), two perforated conical tubes (50 mL) containing either clean mouse bedding (no odor tube) or dirty cat litter (odor tube; from the cat used for stress exposure; containing urine and fur) were affixed to the floor of the open field arena in a cross-over design. In mice not exposed to the predator stress, the ratio of time spent around the odor tube vs. total time spent in either no odor or odor tube is 47%–60% while it is 75%–80% in stressed mice (Toth et al., 2016; Deslauriers et al., 2017), suggesting that stress exposure increases avoidance of predator odor in these mice. Mice were placed in an empty corner and their activity was assessed for 10 min. The number of approaches, the latency of first approach and time spent within a 3-cm radius zone around the tubes were measured using Ethovision Tracking Software.

#### Statistical Analysis

Since the three behavioral paradigms measure generalized avoidance behaviors, we calculated a composite avoidance score (average *z*-score of the number of entries/approaches into, time spent in, and latency of the first entry/approach in the aversive area) as previously described (Toth et al., 2016; Deslauriers et al., 2017). The aversive areas correspond to the center area, the light chamber and the tube containing dirty cat litter in the open field, light-dark box and “trauma-reminder” tests, respectively. This approach allows a more accurate and robust evaluation of the overall long-term effect of the stressor across multiple measures and time points and reduces family-wise error due to multiple testing. This procedure essentially reduces nine avoidance-related variables to 1 composite score. By creating this composite, variables that have relatively little differential meaning [time in open area of OFT vs. time in lit area of light-dark box test (LDT)] cannot be arbitrarily chosen for effects. This composite also prevents the problem of multiple sampling and the need for statistical power to overcome statistical corrections. A Pearson correlation between the nine dependent variables across the three behavioral tests was performed to describe the relationship between the components of the composite avoidance score (Supplementary Table S1). All variables contribute equally to the score, and by creating a composite of assessments over a 1-week period the consistency of the behavioral phenotype is emphasized. In other words, if on one test an animal is highly avoidant, but on other tests is not, it’s overall “PTSD-like phenotype” score will be relatively modest. For each experiment (full and protected exposure), the composite *z-score* was then analyzed using multifactorial analysis of variance (ANOVA) with* COMT* genotype and predator stress as between-subject factors, followed by Sidak *post hoc* comparisons (IBM SPSS Statistics). Since we and others have found that sex modulates COMTval158met SNP associations and COMT functional effects are sex-dependent in both humans and animals (Tunbridge, 2010; Risbrough et al., 2014), we performed the analysis for each sex separately.

### Experiment 2: Effects of Heritable Differences in COMT Function on the Response to a Methylphenidate

#### Experimental Design and Predator Stress Exposure

Male COMT Met/Met and Val/Val mice were assigned to non-stressed or stressed groups (*n* = 92, 9–14 per group). Female mice were dropped from the experiment because they did not show genotype effects on predator stress. The predator stress exposure was performed as described above using the alternate mild “low trauma” protocol (see “Experimental Design and Predator Stress Exposure” section) to avoid ceiling effects of predator stress (Adamec et al., 2006a). Non-stressed mice were handled for 1 min. Starting 7 days after predator stress (or handling for the control group), methylphenidate (3 mg/kg intraperitoneal; Sigma-Aldrich, Saint-Louis, MO, USA) or vehicle (saline) was administered daily until the end of the study. Since differences in COMT enzymatic activity and dopamine tone are observed in the PFC of human and mouse Met/Met and Val/Val carriers (Chen et al., 2004; Tunbridge, 2010; Risbrough et al., 2014), the dose was chosen based on known increases in catecholamines in cortex but not striatum (Koda et al., 2010) with a peak effects 30 min post-injection. Treatment with methylphenidate was performed in the morning or 30 min before behavioral testing. Starting 10 days after predator exposure, several behavioral tests were performed to assess generalized avoidance (OFT, light-dark box, and “trauma-reminder” test, as described above). We also extended the behavioral test battery to include additional measures relevant to PTSD and potential efficacy of methylphenidate, such as working memory (T-maze) and social withdrawal (social preference test, SPT; see experimental design in Figure 1B). The order of testing was established according to previous studies, and we conducted the least disruptive tests first (Contet et al., 2001; Deslauriers et al., 2017).

#### Behavioral Pattern Monitor

To verify that the chronic low-dose treatment did not affect locomotor and exploratory activities, these parameters were assessed in BPM chambers (San Diego Instruments, San Diego, CA, USA; Risbrough et al., 2006) as described above (see “Exploratory Behavior and Locomotor Activity” section). Mice were placed in the BPM chambers (under dark) and transitions (ambulations) were recorded for 60 min.

#### Working Memory

Patients with PTSD often exhibit deficits in working memory, which can also be related to disruption in inhibited processing speed, behavioral inflexibility, attention, and response inhibition (Vasterling et al., 2009). To examine working memory after predator stress, we used the spontaneous alternation task, a common test of spatial working memory in rodents which is sensitive to both cortical catecholamine function and COMT gene function (Lalonde, 2002; Risbrough et al., 2014). For each trial, the mouse begins in the stem of the maze and is allowed to choose one of two arms to explore. Typically rodents choose to explore arms that were not visited in the previous trial, and alternate between arms. This test takes advantage of a rodent’s natural motivation to explore novel vs. familiar environments, and requires the mouse to remember what arm was just visited, while disregarding all other previous trials. The spontaneous alternation test was conducted as previously described (Risbrough et al., 2014) using a black plastic T-maze. The T-maze (81 cm length × 10 cm width × 25 cm height) includes two arms (35 cm length × 10 cm width × 25 cm height) and a start box (8 cm length × 10 cm width) that is separated from the main stem by a horizontal sliding door. Horizontal sliding doors (20 cm high) are also placed at the entrance of each arm. The 1st day, mice were habituated to the maze for 5 min, during which they could freely explore all three arms. The 2nd day, mice were placed in the start box for 30 s before the start of each trial. After 30 s the door was slid open and mice had a free choice into either the left or the right arm. After the mouse made a choice (all four paws in the chosen arm), the arm door was closed and the mice were allowed to explore the arm for 30 s before being removed and replaced in the start box for the next trial. A total of eight trials were completed (seven possible alternations). Percentage of spontaneous alternation was calculated as 100 × (number of alternations/7).

#### Social Approach Test

Social withdrawal is commonly observed in PTSD patients (Gold et al., 2000). Therefore, the preference of the mice for a social stimulus over an inanimate object was assessed. Social approach was tested using a three-chambered box similar to what we have been previously described (Naviaux et al., 2014). Briefly, a Plexiglas box (60 cm length × 60 cm width × 30 cm height) was divided into three equal compartments by Plexiglas partitions containing an opening through which the mice could freely enter the three chambers. The test was conducted in two phases. During the first phase, the test mouse was first allowed to explore the chambers for 10 min. Each of the two outer chambers contained an empty, inverted stainless steel wire cup (Galaxy Cup, Spectrum Diversified Designs, Inc., Streetsboro, OH, USA). During the second phase, the test mouse was briefly removed. An unfamiliar mouse, age- and sex-matched, was placed under one of the wire cups and Lego blocks were placed under the other wire cup. The test mouse was then gently placed back in the arena and given 5 min to explore. The amount of time spent in each chamber was recorded using Ethovision Tracking Software and hand-scored by an experimenter blinded to the condition/treatment. The location (left or right) of the novel object and novel mouse alternated across subjects. SP in percent was calculated as 100 multiplied by the hand-scored time spent exploring (sniffing; bringing its nose in a 2-cm radius zone around the cage) the stranger mouse (t_M_) divided by the sum of the time with stranger plus time with object (t_M_ + t_L_): SP = 100 × [t_M_/(t_M_ + t_L_)].

#### Statistical Analysis

For the three paradigms measuring generalized avoidance behaviors (OFT, LDT, and “trauma-reminder” test), we calculated a composite avoidance score as described above (see “Statistical Analysis” section; Toth et al., 2016; Deslauriers et al., 2017). The composite *z*-score, spontaneous alternation and SP were then analyzed using multifactorial ANOVA with* COMT* genotype, predator stress, and methylphenidate treatment as between-subject factors. For locomotor and exploratory activities assessed in the BPM, repeated measures ANOVAs were conducted with the same between-subject factors and with time (10-min block) as within-subject factor. All ANOVAs were followed by Tukey’s *post hoc* comparisons (IBM SPSS Statistics).

## Results

### Experiment 1: Effects of Heritable Differences in COMT Function on the Response to Low and High Intensity Trauma

#### High Trauma Condition

In males, unprotected predator exposure increased the avoidance composite score (*F*_(1,38)_ = 64.16; *p* < 0.001) independently of genotype as confirmed with *post hoc* comparisons (*p* < 0.001 vs. the corresponding non-stressed group; Figure 2A). *COMT* Val/Val carriers also showed increased avoidance compared to Met/Met carriers (*F*_(1,38)_ = 27.83; *p* < 0.001), as confirmed by *post hoc* test (*p* < 0.001; Figure 2A; Supplementary Table S2). No interaction between *COMT*val158met and stress were detected suggesting additive effects of stress and COMT genotype on avoidance behavior. In females, predator stress resulted in significant increases in avoidance behavior independently of genotype (main effect of stress: *F*_(1,39)_ = 109.90; *p* < 0.001; Sidak *post hoc* comparisons: *p* < 0.001 vs. the corresponding non-stressed group; Figure 2B; Supplementary Table S3).

#### Low Trauma Condition

As in the high trauma condition, *COMT* Val/Val genotype and stress exposure increased avoidance in males (*F*_(1,51)_ = 4.79; *p* < 0.05 and *F*_(1,51)_ = 5.87; *p* < 0.05, respectively; Figure 2C). Sidak *post hoc* analysis showed that predator exposure exerted a greater effect in Val/Val mice, as demonstrated by increased avoidance in response to stress (*p* < 0.05 vs. non-stressed Val/Val mice; Figure 2C; Supplementary Table S4). In this lower stress condition, female Val/Val and stressed mice show increased amount of avoidance (*F*_(1,38)_ = 10.85; *p* < 0.01) independently of the genotype (Figure 2D). *Post hoc* comparisons confirmed the increased avoidance in both stressed Met/Met and Val/Val carriers (*p* < 0.01 compared to the corresponding non-stressed group; Figure 2D; Supplementary Table S5).

### Experiment 2: COMTval158met Modulation of Response to Chronic Treatment With Methylphenidate

#### Avoidance

**Figure 3:**
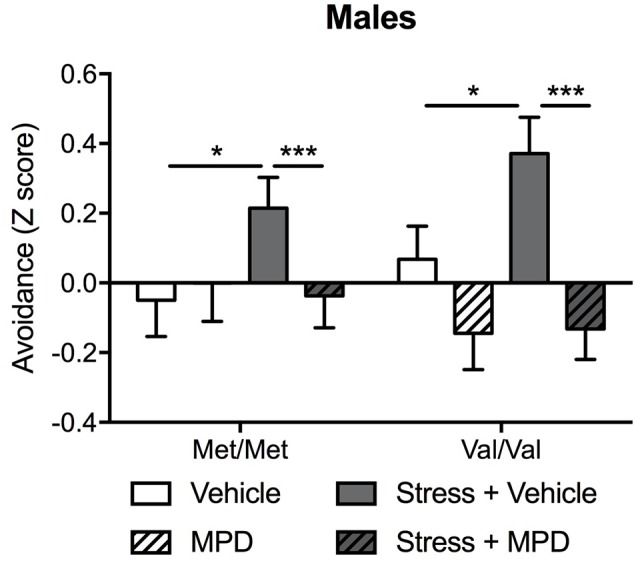
Chronic treatment with methylphenidate (MPD) decreased avoidance behaviors in stressed male mice regardless of their genotype. A composite *z*-score combining all the parameters across the open field, the light-dark box and the “trauma-reminder” (10, 11, and 23 days post stress respectively) testing paradigms was calculated. Data are presented as mean ± SEM. *p < 0.05; ***p < 0.001 (*n* = 9–14 per group).

To examine the overall effects of chronic treatment with methylphenidate on stress-induced PTSD-relevant behaviors, the mild “low trauma” protocol was used for predator stress since it allowed us to prevent ceiling effects of stress that might blur COMTval158met effects (Adamec et al., 2006a). Since the main effect of genotype was found only in males (see above), methylphenidate effects were tested only in male mice. Predator stress increased avoidance behavior which was reversed with methylphenidate treatment (stress × methylphenidate: *F*_(1,84)_ = 4.50; *p* < 0.05). Methylphenidate effects were independent of COMT genotype (Figure 3). *Post hoc* tests confirmed that stressed Met/Met and Val/Val mice treated with methylphenidate exhibited lower avoidance behaviors as compared to the same stressed carriers treated with vehicle (*p* < 0.001; Figure 3). To confirm a genotype effect a two-way ANOVA in vehicle-treated animals alone (genotype × stress) revealed a main effect of genotype (*F*_(1,42)_ = 2.95; *p* < 0.05) and stress (*F*_(1,42)_ = 8.40; *p* < 0.01). *Post hoc* comparisons confirmed that Val/Val carriers showed greater avoidance behavior in response to stress exposure than Met/Met mice (*p* < 0.05 compared to non-stressed Met/Met carriers; Figure 3; Supplementary Table S6).

#### Locomotor Activity

Repeated measures ANOVAs revealed no effect of or interaction with methylphenidate treatment on locomotor activity. A time × genotype interaction was observed (*F*_(1,84)_ = 3.69; *p* < 0.01; Supplementary Figure S2) however no significant differences were detected in *post hoc* analyses at each time point, although Val/Val carriers had slightly higher transitions compared to Met/Met mice at later time points. Univariate ANOVA within each 10-min block did not reveal any main effect or interaction of genotype, stress, or methylphenidate.

#### Working Memory

**Figure 4:**
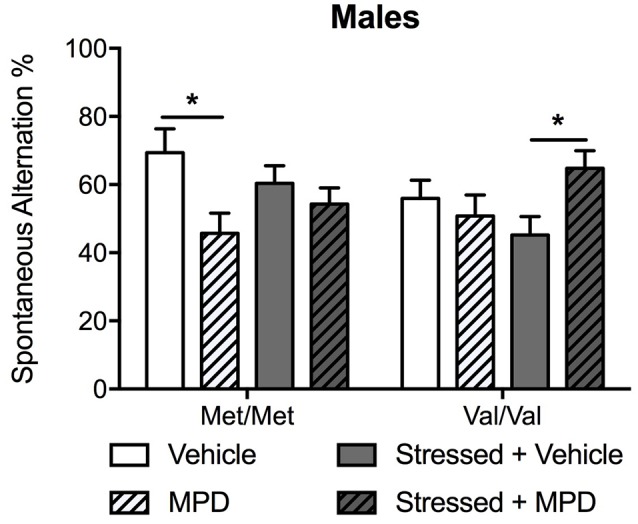
Chronic treatment with MPD had opposite effects on working memory in non-stressed Met/Met carriers and in stressed Val/Val mice. Working memory was determined with the spontaneous alternation assessed in the T-maze 13 days after stress. Data are presented as mean ± SEM in male mice. *p < 0.05 (*n* = 9–14 per group).

To determine the effects of chronic administration of methylphenidate on working memory, spontaneous alternation in the T-maze was assessed. Methylphenidate treatment effects were modulated by genotype (methylphenidate × genotype: *F*_(1,83)_ = 7.61; *p* < 0.01) and stress (methylphenidate × stress: *F*_(1,83)_ = 6.94; *p* = 0.01). *Post hoc* comparisons revealed that methylphenidate decreased spontaneous alternation in non-stressed Met/Met carriers compared to the same non-stressed carriers treated with vehicle (*p* < 0.05; Figure 4). The same treatment had the opposite effect in stressed Val/Val mice by increasing spontaneous alternation (*p* < 0.05 as compared to the same stressed mice treated with vehicle; Figure 4). To replicate our previous observation of COMTval158met modulation of working memory (Risbrough et al., 2014) a two-way ANOVA (genotype × stress) in vehicle-treated mice confirmed that Val/Val carriers exhibited lower spontaneous alternation compared to Met/Met mice (*F*_(1,40)_ = 6.10;   *p* < 0.05,   *post hoc* comparisons,  *p* < 0.05).

#### Social Approach Behavior

**Figure 5:**
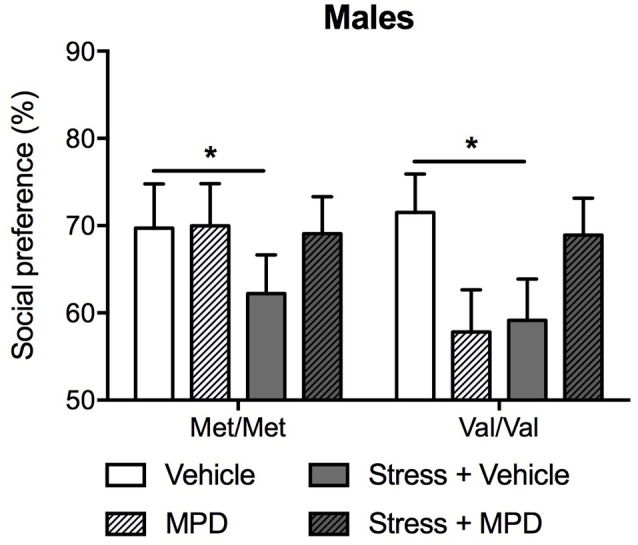
Chronic treatment with MPD prevented stress-induced decrease in social approach in male mice, regardless of their genotype. Social behaviors were assessed in the social approach test 18 days after stress. Data are presented as mean ± SEM. *p < 0.05 (*n* = 9–14 per group).

We then evaluated the effects of chronic treatment of methylphenidate on social approach. Methylphenidate effects on social approach were dependent upon stress exposure (*F*_(1,83)_ = 5.49; *p* < 0.05; Figure 5). Indeed, stressed mice showing decreased social approach, regardless of genotype. Methylphenidate treatment prevented the development of social approach deficits in both stressed Met/Met and Val/Val carriers (Tukey’s *post hoc* comparisons *p* < 0.05 compared to the corresponding non-stressed vehicle-treated group; Figure 5).

## Discussion

The present study aimed to test whether the heritable variance in COMT function produced by the *COMT*val158met SNP modulates response to trauma and to chronic treatment with methylphenidate. In a “humanized” *COMT*val158met mouse line (Risbrough et al., 2014), male Val/Val carriers exhibited higher avoidance behaviors at baseline and in response to a predator stress model of PTSD compared to Met/Met carriers. This phenotype in Val/Val mice was observed across both low and high “trauma” protocols, and in the treatment protocol, underscoring the robustness of the phenotype. In female mice, the stress effects were COMT-independent across both “trauma” protocols, indicating that effects of COMTval158met on avoidance-like traits are sex-dependent. Male Val/Val carriers had lower working memory performance as measured by spontaneous alternation compared to Met/Met carriers. However, no differences were found in SP at baseline or after stress exposure between male Met/Met and Val/Val carriers. Hence in males, COMTval158met modulates avoidance, working memory as well as trauma response, but not social behaviors. Methylphenidate treatment reduced avoidance and increased SP in stressed mice independently of their genotype. However, COMTval158met genotype did modulate methylphenidate effects on spontaneous alternation, decreasing alternation in non-stressed Met/Met mice while increasing alternation in stressed Val/Val mice. These findings suggest that the effects of methylphenidate on avoidance and social behaviors involve COMT-independent mechanisms, and COMT-dependent pathways may modulate the effects of the same treatment on working memory in male mice.

The current study shows that higher COMT function by carrying the Val allele of the *COMT*val158met SNP induced higher avoidance behaviors in male mice. These findings are in line with a meta-analysis (*n* = 2,913) confirming an association between the Val allele and panic disorder in studies with participants of European ancestry (Howe et al., 2016). These results also concur with a previous study linking the Val allele with higher PTSD incidence following mild traumatic brain injury (mTBI) in a predominantly Caucasian sample (Winkler et al., 2017). Together with our preclinical findings, these data support the double-hit hypothesis (gene × environment interaction) of trauma-related disorders (Daskalakis et al., 2013; Smoller, 2016). In female mice, COMT genotype was not associated with different baseline or post-trauma avoidance behavior across either “trauma” protocols. Sex-dependent effects of COMTval158met are in line with previous studies reported a strong sexually dimorphic effect of COMT on brain and behavior (Tunbridge and Harrison, 2010). However, stronger effects of COMT inhibition were found with tolcapone in female rats (Laatikainen et al., 2013) or in COMT-deficient female mice (Gogos et al., 1998) compared to male rodents. These discrepancies with our findings may be due to complete inhibition of COMT function, whereas our COMT Met/Met mice exhibit a 50% inhibition of COMT function compared to Val/Val carriers (Risbrough et al., 2014). Animal studies have also shown that estrogen enhances dopamine activity in the PFC (Jacobs and D’Esposito, 2011), suggesting that estrogen may mitigate COMTval158met effects. Nevertheless, further work characterizing the effects of estrogen on catecholamine signaling in our model needs to be conducted to understand the mechanisms underlying the sex-dependent effects of the COMTval158met. In the spontaneous alternation test, we replicated past observations of low alternation in Val/Val mice compared to Met/Met mice (Risbrough et al., 2014), which is in line with some human studies reporting poorer working memory performance in of Val/Val carriers (Giakoumaki et al., 2008; Costa Dde et al., 2016; Geller et al., 2017; Miskowiak et al., 2017). We observed no effects of COMTval158met genotype on SP, which concurs with a previous clinical study showing no association between COMTval158met and social functioning in patients with schizophrenia or bipolar disorder (Goghari and Sponheim, 2008).

COMTval158met effects on response to a trauma could be *via* modulation of cortical catecholamine signaling both during and after the trauma. COMT has a significant contribution to catecholamine signaling in the cortex because of relatively low dopamine transporter expression (Harrison and Tunbridge, 2008). The PFC modulates fear responses through reciprocal inputs to the amygdala and is involved in multiple forms of fear inhibition including fear extinction, which can be impaired in PTSD patients (Bremner, 2005; Gamo and Arnsten, 2011). The *COMT*val158met SNP alters catecholamine signaling pathways in the PFC, with Val/Val carriers exhibiting higher COMT activity in this circuit compared to Met/Met carriers. Subsequently, mouse and human Val/Val carriers have lower dopamine tone than Met/Met carriers (Tunbridge and Harrison, 2010; Risbrough et al., 2014). There is some evidence that lower cortical dopamine tone can promote anxiety responses. Chronic stress reduces dopamine levels in the mesocortical region in rodents (Burke and Miczek, 2014), and cortical dopamine depletion is anxiogenic in rodents (Sullivan et al., 2014). Most pertinent to the current study, exposure to predator odor elevates dopamine turnover in the medial PFC (mPFC), which is correlated with increased avoidance (Morrow et al., 2000). Hence it is possible that the combination of stress-induced reductions in cortical dopamine signaling along with higher dopamine catabolism in Val/Val carriers underlies the modulation of *COMT*val158met on enduring stress response (Arnsten et al., 2015). It should also be noted that a potential role of noradrenergic signaling in *COMT*val158met effects on stress responses should not be excluded (Arnsten et al., 2015). Indeed, since the COMTval158met affects noradrenergic pathways (Tunbridge and Harrison, 2010), it would be interesting to investigate the effects of COMTval158met and predator stress on hyperarousal and corticosterone levels. Further studies using this “humanized” *COMT* mouse line exposed to predator stress will be necessary to confirm the underlying mechanisms of *COMT*val158met modulation of enduring response to trauma.

We also investigated the effects of chronic treatment with methylphenidate in the predator stress model of PTSD in males and asked if COMTval158met modulated treatment response. Acute low doses of methylphenidate (<5 mg/kg) reduce anxiety-like behaviors (Koike et al., 2009; Mioranzza et al., 2010), whereas high doses (>7 mg/kg) have anxiogenic effects (Ihne et al., 2012). We chose a dose of MPD that selectively increases catecholamine release in cortex while having little effect in striatum in mice, to specifically target circuits that are most sensitive to COMT variation (Chen et al., 2004; Tunbridge, 2010; Risbrough et al., 2014). Higher doses of MPD induce increases in striatal dopamine and concomitant stimulant activity, which could also confound activity-based measures used in this study (Koda et al., 2010). Here, we confirmed that this dosing regimen did not induce changes in locomotor and exploratory behavior. This dose is also very similar to effective doses of MPD in rat models of PTSD (Avital et al., 2011; Zubedat et al., 2015).

Here, the treatment was tested only in males since females did not show significant *COMT* variant effects on predator stress. Methylphenidate treatment ameliorated both stress-induced avoidance and reduction in SP. These data are in line with other reports of methylphenidate efficacy in animal models of PTSD (Aga-Mizrachi et al., 2014; Ritov and Richter-Levin, 2017) and social approach deficits (Aga-Mizrachi et al., 2014; Gill et al., 2014). It is also in line with reports of efficacy in PTSD patients (McAllister et al., 2016) and for social functioning in ADHD (Abikoff et al., 2004). COMTval158met did not modify treatment effects in these tests, suggesting these treatment effects are independent of heritable differences in COMT function. There are a number of potential mechanisms underlying the effects of methylphenidate to treat enduring avoidance and other stress-related symptoms after trauma. In rodents, chronic administration of low-dose methylphenidate increases attention in several cognitive tasks *via* activation of α_2_ adrenergic and D1 dopaminergic receptors in the rat PFC (Spencer et al., 2015). Chronic low-dose methylphenidate also restores stress-induced alterations in PFC dendritic spine densities and impairments in NMDAR function (Zehle et al., 2007; Cheng et al., 2014). It also amplifies long-term potentiation (LTP) in rat hippocampal CA1 area *via* the activation of β-adrenergic and D1/D5 dopaminergic receptors (Rozas et al., 2015). As stated above, PFC and hippocampal dysfunctions are consistent “biophenotypes” in PTSD, and there is also mounting evidence for glutamatergic disruption in PTSD (Averill et al., 2017; Deslauriers et al., 2018). Nevertheless, further work needs to be conducted to investigate the exact therapeutic mechanisms of chronic low-dose methylphenidate, as well as its sex-dependent effects in the context of PTSD.

In line with the COMT-dependent effects of methylphenidate on working memory reported here, treatment with COMT inhibitor tolcapone improves working memory in human Val carriers but reduces the high working memory performance in Met carriers (Giakoumaki et al., 2008). These data support the hypothesis that COMTval158met modulates the “inverted U” effects of dopamine signaling on PFC functions such as working memory. No significant association was found between COMTval158met and response to methylphenidate treatment in adolescent and adult patients with ADHD (Contini et al., 2012; Unal et al., 2016). However, a meta-analysis (*n* = 889) reported increased response to methylphenidate in ADHD children carrying the Val/Val genotype (Myer et al., 2018), suggesting that the COMT-dependent effects of methylphenidate may be age-dependent. Here, methylphenidate exerted COMT-dependent effects on working memory, but not avoidance and social behaviors in mice. Together with the clinical findings reported above, these findings suggest that the COMT-dependent effects of methylphenidate may be specific to attention and memory.

The present study showed an increased response to predator-induced trauma in male Val/Val mice. Furthermore, methylphenidate reversed the predator stress-induced avoidance behaviors and social withdrawal in males regardless of genotype while methylphenidate effects on cognition were dependent upon COMTval158met genotype. These results suggest that individual differences in catecholamine signaling may modulate response to a trauma and support the double-hit gene × environment hypothesis of PTSD. These findings also support recent human findings that methylphenidate might be a therapeutic alternative for PTSD patients (McAllister et al., 2016) and suggest that methylphenidate effects anxiety vs. cognitive symptoms in males may be modulated through COMT-independent and dependent mechanisms respectively. Nevertheless, further work needs to be conducted to investigate the COMT variant-specific response to methylphenidate in females.

## Ethics Statement

The experiments were conducted in accordance with the Principles of Laboratory Animal Care, National Institutes of Health guidelines, as approved by the University of California, San Diego, San Diego, CA, USA.

## Author Contributions

VR designed the experiment, and MT conducted the experiments using the full exposure to predator stress. JD performed all the other experiments, analyzed and interpreted the data. XZ developed the “humanized” COMT mouse line. JD and VR wrote the manuscript, and MT and XZ provided critical feedback.

## Conflict of Interest Statement

The authors declare that the research was conducted in the absence of any commercial or financial relationships that could be construed as a potential conflict of interest.

## Supplementary Material

FIGURE S1 No difference on baseline locomotor activity was found across all groups in all experiments. Locomotor activity (counts) was assessed **(A–D)** for Experiment 1 (COMTval158met modulation of response to low and high trauma; **A–D**) and Experiment 2 (COMTval158met modulation of response to methylphenidate; **E**) were assessed in the behavioral pattern monitor 1 week before stress. Data are presented as mean ± SEM (*n* = 8–14 per group).

FIGURE S2 Heritable differences in COMT function, stress, and methylphenidate did not affect locomotor activity in male mice. Locomotor activity (transitions) was assessed in the behavioral pattern monitor 10 days after stress. Data are presented as mean ± SEM for non-stressed and stressed Met/Met carriers **(A,B)**, as well as for non-stressed and stressed Val/Val mice **(C,D)** (*n* = 9–14 per group).

TABLE S1 Pearson correlation matrix of all variables included in the composite avoidance score across all three experiments (unprotected and protected predator exposures, and chronic methylphenidate experiment). Since the three behavioral paradigms measure generalized avoidance behaviors, we calculated a composite avoidance score (average *z*-score of the number of entries/approaches into, time spent in, and latency of the first entry/approach in the aversive area) as previously described (Toth et al., 2016; Deslauriers et al., 2017). The aversive areas correspond to the center area, the light chamber and the tube containing dirty cat litter in the open field, light-dark box and “trauma-reminder” tests, respectively (*n* = 187).

TABLE S2 Avoidance behavior in the open field, light-dark box and trauma-reminder tests following full predator exposure (high trauma) in male mice. Data are presented as mean ± SEM for the number of entries/approaches, time spent and latency of first entry/approach in the aversive area across all the behavioral tests. The aversive areas are the center area, the lit chamber and the odor tube in the open field, light-dark box and trauma-reminder tests, respectively. Two-way ANOVAs (genotype × stress) revealed a main effect of stress in the open field test (*F*_(1,38)_ = 13.76; *p* < 0.01), with decreased time spent in the center area in predator-exposed mice. Stress also increased the latency of first approach to the cat odor tube in the trauma-reminder test (*F*_(1,38)_ = 4.49; *p* < 0.05). ***p* < 0.01 vs. non-stressed Met/Met mice following Sidak *post hoc* test.

TABLE S3 Avoidance behavior in the open field, light-dark box and trauma-reminder tests following full predator exposure (high trauma) in female mice. Data are presented as mean ± SEM for the number of entries/approaches, time spent and latency of first entry/approach in the aversive area across all the behavioral tests. The aversive areas are the center area, the lit chamber and the odor tube in the open field, light-dark box and trauma-reminder tests, respectively. Two-way ANOVAs (genotype × stress) revealed a main effect of stress in the open field test, with decreased number of entries time spent in the center area (*F*_(1,39)_ = 10.57; *p* < 0.01 and *F*_(1,39)_ = 8.23; *p* < 0.01, respectively), and increased latency of first entry in the center arena (*F*_(1,39)_ = 7.70; *p* < 0.01) in predator-exposed mice. In the light-dark box, stressed mice exhibited reduced number of entries in the lit chamber (*F*_(1,39)_ = 4.76; *p* < 0.05). **p* < 0.05 vs. non-stressed Met/Met mice; ^#^*p* < 0.05 vs. non-stressed Val/Val mice following Sidak *post hoc* test.

TABLE S4 Avoidance behavior in the open field, light-dark box and trauma-reminder tests following protected predator exposure (low trauma) in male mice. Data are presented as mean × SEM for the number of entries/approaches, time spent and latency of first entry/approach in the aversive area across all the behavioral tests. The aversive areas are the center area, the lit chamber and the odor tube in the open field, light-dark box and trauma-reminder tests, respectively. Two-way ANOVAs (genotype × stress) revealed a main effect of stress in the open field test (*F*_(1,51)_ = 2.66; *p* < 0.001), with increased latency to first entry to center area in predator-exposed mice. Also, the genotype decreased the time spent in the lit chamber of the light-dark box (*F*_(1,51)_ = 10.79; *p* < 0.01) and around to cat odor tube in the trauma-reminder test (*F*_(1,51)_ = 11.04; *p* < 0.01). The COMT genotype also increased the latency to the first approach to the odor tube in the trauma-reminder test (*F*_(1,51)_ = 8.73; *p* < 0.01). **p* < 0.05 vs. non-stressed Met/Met mice; ^###^*p* < 0.001 vs. non-stressed Val/Val group following Sidak *post hoc* test.

TABLE S5 Avoidance behavior in the open field, light-dark box and trauma-reminder tests following protected predator exposure (low trauma) in female mice. Data are presented as mean ± SEM for the number of entries/approaches, time spent and latency of first entry/approach in the aversive area across all the behavioral tests. The aversive areas are the center area, the lit chamber and the odor tube in the open field, light-dark box and trauma-reminder tests, respectively. Two-way ANOVAs (genotype × stress) revealed a main effect of stress in the open field and the trauma-reminder tests, mainly on latency to first entry to center area (*F*_(1,51)_ = 9.72; *p* < 0.01) and the latency to first approach to the odor tube (*F*_(1,51)_ = 4.41; *p* < 0.05), respectively. Also, the genotype interacted with stress (main effect of genotype: *F*_(1,51)_ = 4.32, *p* < 0.05; genotype stress: *F*_(1,51)_ = 5.67, *p* < 0.05) in the trauma-reminder stress, with Val/Val carriers exposed to a predator exhibiting lower time spent around the cat odor tube. **p* < 0.05 vs. non-stressed Met/Met mice; ^#^*p* < 0.05 vs. non-stressed Val/Val group following Sidak *post hoc* test.

TABLE S6 Avoidance behavior in the open field, light-dark box and trauma-reminder tests following protected predator exposure and chronic treatment with methylphenidate in male mice. Data are presented as mean ± SEM for the number of entries/approaches, time spent and latency of first entry/approach in the aversive area across all the behavioral tests. The aversive areas are the center area, the lit chamber and the odor tube in the open field, light-dark box and trauma-reminder tests, respectively. In the open field test, a three-way ANOVAs (genotype × stress × methylphenidate) revealed a main effect of methylphenidate (*F*_(1,84)_ = 12.87; *p* < 0.001; genotype × methylphenidate: *F*_(1,84)_ = 4.41; *p* < 0.05) on the number of entries in center area, with increased number of entries in the center area in Val/Val mice treated with methylphenidate vs. corresponding group treated with vehicle. Methylphenidate also decreased the time spent in the center area (*F*_(1,84)_ = 5.48; *p* < 0.05), and a genotype × stress interaction (*F*_(1,84)_ = 4.53; *p* < 0.05) was found on the latency of first entry in the center area. In the trauma-reminder test, methylphenidate increased the number of approaches to cat odor tube (*F*_(1,84)_ = 26.19; *p* < 0.001). **p* < 0.05 and ***p* < 0.01 vs. corresponding group treated with vehicle following Tukey’s *post hoc* test.
