# Supplementary figures and images for: Heritable Differences in Catecholamine Signaling Modulate Susceptibility to Trauma and Response to Methylphenidate Treatment: Relevance for PTSD
Source: Front Behav Neurosci. 2019 May 17;13:111. doi: 10.3389/fnbeh.2019.00111 (PMC6534065; doi:10.3389/fnbeh.2019.00111)

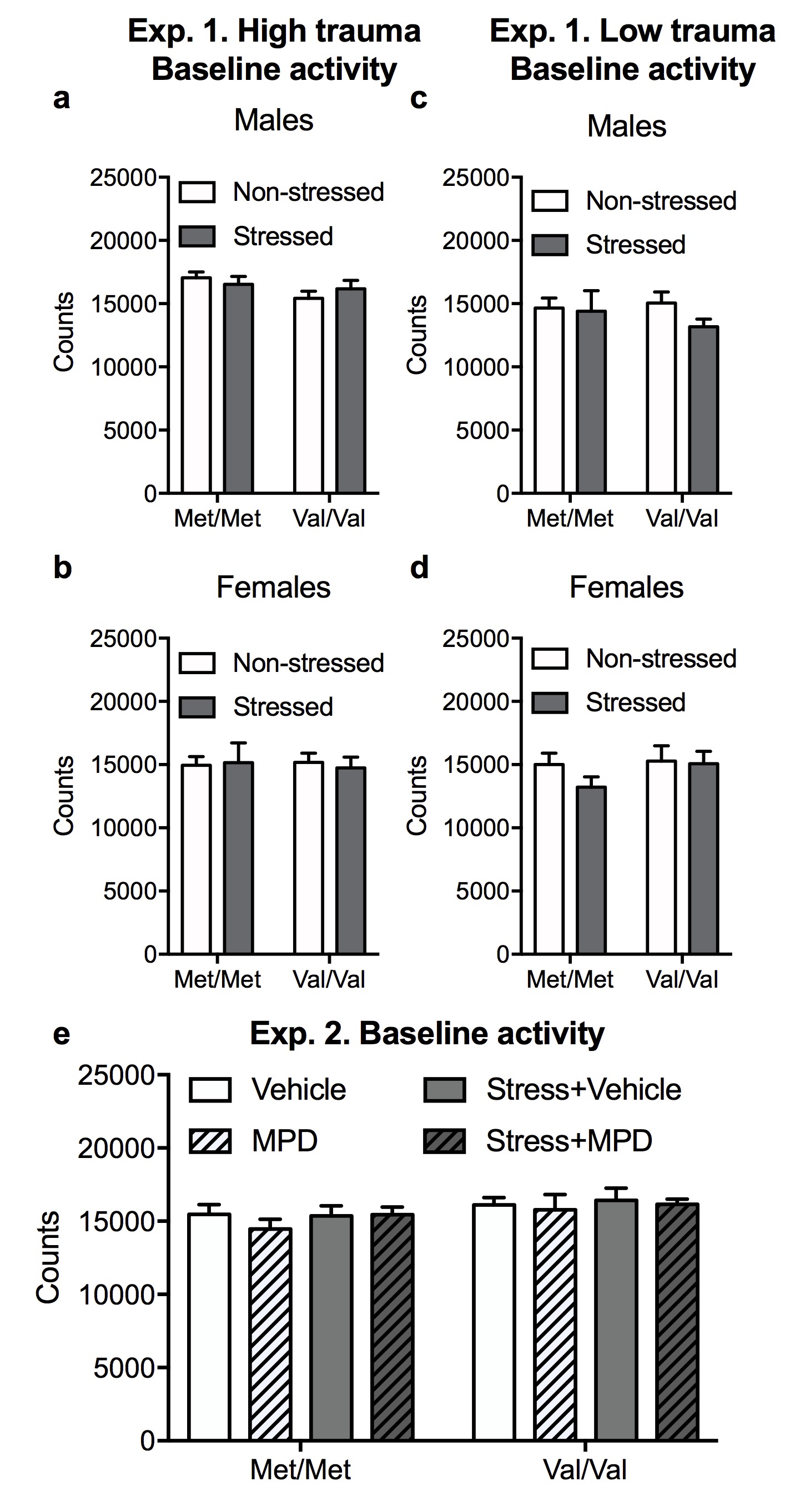

Supplement: FIGURE S1 — No difference on baseline locomotor activity was found across all groups in all experiments. Locomotor activity (counts) was assessed (A–D) for Experiment 1 (COMTval158met modulation of response to low and high trauma; A–D) and Experiment 2 (COMTval158met modulation of response to methylphenidate; E) were assessed in the behavioral pattern monitor 1 week before stress. Data are presented as mean ± SEM (n = 8–14 per group). [file Image_1.JPEG]

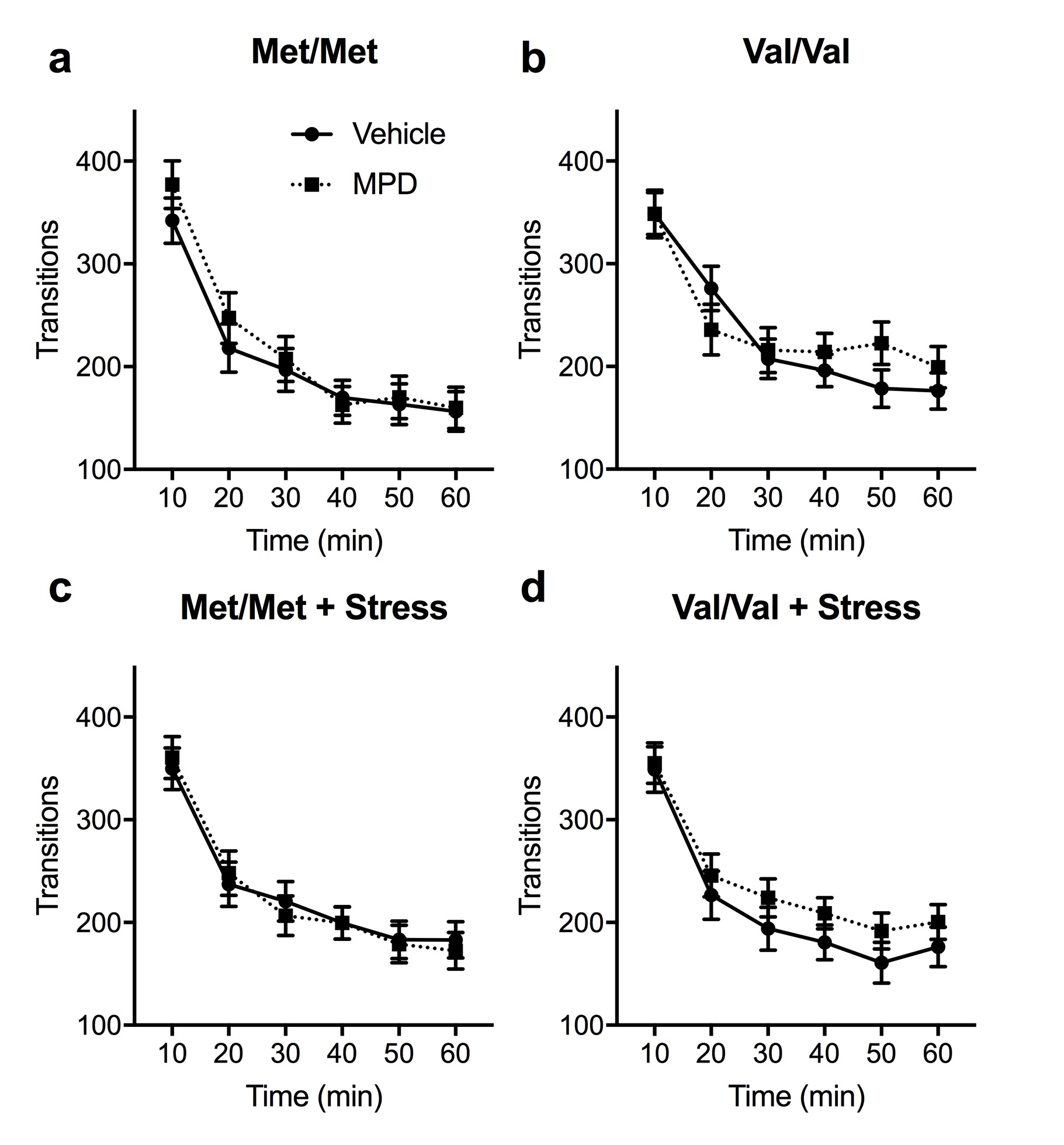

Supplement: FIGURE S2 — Heritable differences in COMT function, stress, and methylphenidate did not affect locomotor activity in male mice. Locomotor activity (transitions) was assessed in the behavioral pattern monitor 10 days after stress. Data are presented as mean ± SEM for non-stressed and stressed Met/Met carriers (A,B), as well as for non-stressed and stressed Val/Val mice (C,D) (n = 9–14 per group). [file Image_2.JPEG]
