# Supplementary material for: Heritable Differences in Catecholamine Signaling Modulate Susceptibility to Trauma and Response to Methylphenidate Treatment: Relevance for PTSD
Source: Front Behav Neurosci. 2019 May 17;13:111. doi: 10.3389/fnbeh.2019.00111 (PMC6534065; doi:10.3389/fnbeh.2019.00111)
Supplement: TABLE S1 — Pearson correlation matrix of all variables included in the composite avoidance score across all three experiments (unprotected and protected predator exposures, and chronic methylphenidate experiment). Since the three behavioral paradigms measure generalized avoidance behaviors, we calculated a composite avoidance score (average z-score of the number of entries/approaches into, time spent in, and latency of the first entry/approach in the aversive area) as previously described (Toth et al., 2016; Deslauriers et al., 2017). The aversive areas correspond to the center area, the light chamber and the tube containing dirty cat litter in the open field, light-dark box and “trauma-reminder” tests, respectively (n = 187). [file Table_1.DOC]

**Supplemental table 1.** Pearson correlation matrix of all variables included in the composite avoidance score across all three experiments (unprotected and protected predator exposures, and chronic methylphenidate experiment)

|  | | Open Field | | | Light-dark box | | | Trauma-reminder test | | |
| --- | --- | --- | --- | --- | --- | --- | --- | --- | --- | --- |
|  |  | Frequency | Time | Latency | Frequency | Time | Latency | Frequency | Time | Latency |
| Open Field | Frequency |  | **0.496 (*p*<0.001)** | **0.217**  **(*p*<0.01)** | 0.110  (*p*=0.13) | -0.018 (*p*=0.81) | -0.037 (*p*=0.61) | **0.427 (*p*<0.001)** | -0.122 (*p*=0.10) | -0.095 (*p*=0.20) |
|  | Time | **0.496 (*p*<0.001)** |  | **0.327 (*p*<0.001)** | 0.070 (*p*=0.33) | 0.080  (*p*=0.27) | -0.048  (*p*=0.51) | 0.065  (*p*=0.37) | -0.092  (*p*=0.21) | -0.046  (*p*=0.53) |
|  | Latency | **0.217**  **(*p*<0.01)** | **0.327 (*p*<0.001)** |  | 0.043 (*p*=0.55) | 0.080 (*p*=0.27) | 0.045 (*p*=0.54) | **0.163 (*p*<0.05)** | 0.132 (*p*=0.07) | 0.113 (*p*=0.12) |
| Light-dark box | Frequency | 0.110  (*p*=0.13) | 0.070 (*p*=0.33) | 0.043 (*p*=0.55) |  | **0.194**  **(*p*<0.01)** | **0.233**  **(*p*<0.001)** | 0.019 (*p*=0.80) | -0.023 (*p*=0.75) | 0.012 (*p*=0.87) |
|  | Time | -0.018 (*p*=0.81) | 0.080  (*p*=0.27) | 0.080 (*p*=0.27) | **0.194**  **(*p*<0.01)** |  | **0.248**  **(*p*<0.001)** | -0.010 (*p*=0.89) | **0.211**  **(*p*<0.01)** | **0.147**  **(*p*<0.05)** |
|  | Latency | -0.037 (*p*=0.61) | -0.048  (*p*=0.51) | 0.045 (*p*=0.54) | **0.233**  **(*p*<0.001)** | **0.248**  **(*p*<0.001)** |  | -0.023 (*p*=0.75) | -0.015 (*p*=0.84) | 0.104 (*p*=0.16) |
| Trauma-reminder test | Frequency | **0.427 (*p*<0.001)** | 0.065  (*p*=0.37) | **0.163 (*p*<0.05)** | 0.019 (*p*=0.80) | -0.010 (*p*=0.89) | -0.023 (*p*=0.75) |  | **0.358 (*p*<0.001)** | **0.160 (*p*<0.05)** |
|  | Time | -0.122 (*p*=0.10) | -0.092  (*p*=0.21) | 0.132 (*p*=0.07) | -0.023 (*p*=0.75) | **0.211**  **(*p*<0.01)** | -0.015 (*p*=0.84) | **0.358 (*p*<0.001)** |  | **0.178 (*p*<0.05)** |
|  | Latency | -0.095 (*p*=0.20) | -0.046  (*p*=0.53) | 0.113 (*p*=0.12) | 0.012 (*p*=0.87) | **0.147**  **(*p*<0.05)** | 0.104 (*p*=0.16) | **0.160 (*p*<0.05)** | **0.178 (*p*<0.05)** |  |

Since the three behavioral paradigms measure generalized avoidance behaviors, we calculated a composite avoidance score (average z-score of the number of entries/approaches into, time spent in, and latency of the first entry/approach in the aversive area) as previously described (Deslauriers et al., 2017b; Toth et al., 2015). The aversive areas correspond to the center area, the light chamber and the tube containing dirty cat litter in the open field, light-dark box and “trauma-reminder” tests, respectively (*n*=187).

**Supplemental table 2. Avoidance behavior in the open field, light-dark box and trauma-reminder tests following full predator exposure (high trauma) in male mice.**

| **Open Field** | | | |
| --- | --- | --- | --- |
| **Group** | **Number of entries** | **Time spent** | **Latency of first entry** |
| Non-stressed Met/Met | 75.71 ± 9.73 | 150.59 ± 12.46 | 13.01 ± 7.26 |
| Stressed Met/Met | 58.00 ± 7.83 | 93.71 ± 11.20** | 12.07 ± 3.42 |
| Non-stressed Val/Val | 60.00 ± 5.29 | 129.11 ± 8.56 | 13.44 ± 5.57 |
| Stressed Val/Val | 54.00 ± 4.58 | 100.94 ± 9.99 | 31.84 ± 13.94 |
| **Light-dark box** | | | |
| **Group** | **Number of entries** | **Time spent** | **Latency of first entry** |
| Non-stressed Met/Met | 23.07 ± 1.68 | 202.26 ± 13.87 | 12.19 ± 6.20 |
| Stressed Met/Met | 21.64 ± 2.23 | 213.53 ± 15.23 | 26.53 ± 12.93 |
| Non-stressed Val/Val | 25.00 ± 2.59 | 216.12 ± 18.60 | 7.10 ± 2.31 |
| Stressed Val/Val | 21.00 ± 2.38 | 201.72 ± 20.10 | 21.49 ± 8.54 |
| **Trauma-reminder test** | | | |
| **Group** | **Number of approaches** | **Time spent** | **Latency of first approach** |
| Non-stressed Met/Met | 30.50 ± 3.42 | 111.40 ± 7.60 | 5.02 ± 1.02 |
| Stressed Met/Met | 28.00 ± 2.90 | 129.20 ± 5.07 | 8.05 ± 1.69 |
| Non-stressed Val/Val | 22.75 ± 1.81 | 92.77 ± 7.22 | 3.89 ± 1.13 |
| Stressed Val/Val | 23.50 ± 2.79 | 88.75 ± 10.07 | 9.00 ± 3.31 |

Data are presented as mean ± SEM for the number of entries/approaches, time spent and latency of first entry/approach in the aversive area across all the behavioral tests. The aversive areas are the center area, the lit chamber and the odor tube in the open field, light-dark box and trauma-reminder tests, respectively. Two-way ANOVAs (genotype × stress) revealed a main effect of stress in the open field test (F_1,38_ = 13.76; *p* < 0.01), with decreased time spent in the center area in predator-exposed mice. Stress also increased the latency of first approach to the cat odor tube in the trauma-reminder test (F_1,38_ = 4.49; *p* < 0.05). ***p* < 0.01 vs. non-stressed Met/Met mice following Sidak *post hoc* test.

**Supplemental table 3.** Avoidance behavior in the open field, light-dark box and trauma-reminder tests following full predator exposure (high trauma) in female mice.

| **Open Field** | | | |
| --- | --- | --- | --- |
| **Group** | **Number of entries** | **Time spent** | **Latency of first entry** |
| Non-stressed Met/Met | 79.33 ± 13.92 | 118.55 ± 15.39 | 6.31 ± 1.89 |
| Stressed Met/Met | 48.33 ± 8.06 | 76.57 ± 13.71* | 27.47 ± 7.53 |
| Non-stressed Val/Val | 81.18 ± 9.06 | 100.12 ± 9.27 | 10.91 ± 3.87 |
| Stressed Val/Val | 50.57 ± 6.94# | 76.32 ± 8.55 | 16.63 ± 4.52 |
| **Light-dark box** | | | |
| **Group** | **Number of entries** | **Time spent** | **Latency of first entry** |
| Non-stressed Met/Met | 27.67 ± 1.55 | 204.83 ± 20.75 | 7.92 ± 2.91 |
| Stressed Met/Met | 23.78 ± 2.43 | 163.59 ± 10.44 | 16.79 ± 4.80 |
| Non-stressed Val/Val | 24.36 ± 1.48 | 167.20 ± 7.92 | 6.95 ± 2.85 |
| Stressed Val/Val | 20.21 ± 1.72 | 161.49 ± 11.92 | 10.84 ± 3.22 |
| **Trauma-reminder test** | | | |
| **Group** | **Number of approaches** | **Time spent** | **Latency of first approach** |
| Non-stressed Met/Met | 28.56 ± 3.76 | 69.48 ± 11.46 | 3.35 ± 1.27 |
| Stressed Met/Met | 28.44 ± 3.06 | 85.66 ± 8.70 | 5.08 ± 3.53 |
| Non-stressed Val/Val | 34.55 ± 3.65 | 86.93 ± 7.37 | 5.32 ± 1.37 |
| Stressed Val/Val | 26.43 ± 3.77 | 68.41 ± 8.77 | 7.31 ± 2.53 |

Data are presented as mean ± SEM for the number of entries/approaches, time spent and latency of first entry/approach in the aversive area across all the behavioral tests. The aversive areas are the center area, the lit chamber and the odor tube in the open field, light-dark box and trauma-reminder tests, respectively. Two-way ANOVAs (genotype × stress) revealed a main effect of stress in the open field test, with decreased number of entries time spent in the center area (F_1,39_ = 10.57; *p* < 0.01 and F_1,39_ = 8.23; *p* < 0.01, respectively), and increased latency of first entry in the center arena (F_1,39_ = 7.70; *p* < 0.01) in predator-exposed mice. In the light-dark box, stressed mice exhibited reduced number of entries in the lit chamber (F_1,39_ = 4.76; *p* < 0.05). **p* < 0.05 vs. non-stressed Met/Met mice; #*p* < 0.05 vs. non-stressed Val/Val mice following Sidak *post hoc* test.

**Supplemental table 4. Avoidance behavior in the open field, light-dark box and trauma-reminder tests following protected predator exposure (low trauma) in male mice.**

| **Open Field** | | | |
| --- | --- | --- | --- |
| **Group** | **Number of entries** | **Time spent** | **Latency of first entry** |
| Non-stressed Met/Met | 70.64 ± 4.97 | 116.00 ± 12.82 | 2.54 ± 0.89 |
| Stressed Met/Met | 60.82 ± 2.65 | 109.90 ± 6.32 | 9.34 ± 1.87* |
| Non-stressed Val/Val | 66.20 ± 3.27 | 123.30 ± 10.68 | 1.72 ± 0.59 |
| Stressed Val/Val | 67.78 ± 3.34 | 117.00 ± 6.30 | 13.79 ± 1.79^###^ |
| **Light-dark box** | | | |
| **Group** | **Number of entries** | **Time spent** | **Latency of first entry** |
| Non-stressed Met/Met | 25.90 ± 3.94 | 462.10 ± 43.85 | 3.94 ± 2.38 |
| Stressed Met/Met | 25.13 ± 6.75 | 500.80 ± 30.85 | 1.05 ± 0.59 |
| Non-stressed Val/Val | 28.31 ± 3.97 | 350.20 ± 38.51 | 7.40 ± 2.73 |
| Stressed Val/Val | 24.88 ± 7.68 | 368.60 ± 30.55 | 6.24 ± 2.24 |
| **Trauma-reminder test** | | | |
| **Group** | **Number of approaches** | **Time spent** | **Latency of first approach** |
| Non-stressed Met/Met | 28.36 ± 1.90 | 111.40 ± 7.60 | 5.11 ± 1.73 |
| Stressed Met/Met | 28.73 ± 1.65 | 129.20 ± 5.07 | 1.05 ± 0.59 |
| Non-stressed Val/Val | 30.13 ± 2.54 | 92.77 ± 7.22 | 9.29 ± 2.12 |
| Stressed Val/Val | 26.65 ± 1.75 | 88.75 ± 10.07 | 9.48 ± 2.83 |

Data are presented as mean ± SEM for the number of entries/approaches, time spent and latency of first entry/approach in the aversive area across all the behavioral tests. The aversive areas are the center area, the lit chamber and the odor tube in the open field, light-dark box and trauma-reminder tests, respectively. Two-way ANOVAs (genotype × stress) revealed a main effect of stress in the open field test (F_1,51_ = 2.66; *p* < 0.001), with increased latency to first entry to center area in predator-exposed mice. Also, the genotype decreased the time spent in the lit chamber of the light-dark box (F_1,51_ = 10.79; *p* < 0.01) and around to cat odor tube in the trauma-reminder test (F_1,51_ = 11.04; *p* < 0.01). The *COMT* genotype also increased the latency to the first approach to the odor tube in the trauma-reminder test (F_1,51_ = 8.73; *p* < 0.01). **p* < 0.05 vs. non-stressed Met/Met mice; ###*p* < 0.001 vs. non-stressed Val/Val group following Sidak *post hoc* test.

**Supplemental table 5. Avoidance behavior in the open field, light-dark box and trauma-reminder tests following protected predator exposure (low trauma) in female mice.**

| **Open Field** | | | |
| --- | --- | --- | --- |
| **Group** | **Number of entries** | **Time spent** | **Latency of first entry** |
| Non-stressed Met/Met | 65.33 ± 4.00 | 111.90 ± 8.03 | 12.86 ± 3.50 |
| Stressed Met/Met | 61.79 ± 3.75 | 107.90 ± 7.50 | 4.02 ± 1.12* |
| Non-stressed Val/Val | 64.77 ± 4.39 | 110.40 ± 8.19 | 11.79 ± 2.93 |
| Stressed Val/Val | 61.62 ± 5.36 | 94.33 ± 6.66 | 4.99 ± 1.26 |
| **Light-dark box** | | | |
| **Group** | **Number of entries** | **Time spent** | **Latency of first entry** |
| Non-stressed Met/Met | 20.60 ± 4.41 | 425.20 ± 40.46 | 1.89 ± 0.77 |
| Stressed Met/Met | 23.14 ± 2.77 | 352.70 ± 30.13 | 10.71 ± 3.29 |
| Non-stressed Val/Val | 27.58 ± 2.33 | 377.60 ± 29.42 | 6.67 ± 2.49 |
| Stressed Val/Val | 29.23 ± 2.90 | 374.60 ± 28.03 | 7.15 ± 1.86 |
| **Trauma-reminder test** | | | |
| **Group** | **Number of approaches** | **Time spent** | **Latency of first approach** |
| Non-stressed Met/Met | 27.67 ± 2.16 | 86.25 ± 6.48 | 6.37 ± 1.87 |
| Stressed Met/Met | 28.87 ± 4.30 | 92.73 ± 13.23 | 9.38 ± 2.29 |
| Non-stressed Val/Val | 27.46 ± 2.67 | 89.47 ± 11.00 | 4.89 ± 1.87 |
| Stressed Val/Val | 20.38 ± 3.18 | 45.31 ± 10.56# | 11.77 ± 3.08 |

Data are presented as mean ± SEM for the number of entries/approaches, time spent and latency of first entry/approach in the aversive area across all the behavioral tests. The aversive areas are the center area, the lit chamber and the odor tube in the open field, light-dark box and trauma-reminder tests, respectively. Two-way ANOVAs (genotype × stress) revealed a main effect of stress in the open field and the trauma-reminder tests, mainly on latency to first entry to center area (F_1,51_ = 9.72; *p* < 0.01) and the latency to first approach to the odor tube (F_1,51_ = 4.41; *p* <0.05), respectively. Also, the genotype interacted with stress (main effect of genotype: F_1,51_ = 4.32, p < 0.05; genotype × stress: F_1,51_ = 5.67, *p* < 0.05) in the trauma-reminder stress, with Val/Val carriers exposed to a predator exhibiting lower time spent around the cat odor tube. **p* < 0.05 vs. non-stressed Met/Met mice; #*p* < 0.05 vs. non-stressed Val/Val group following Sidak *post hoc* test.

**Supplemental table 6. Avoidance behavior in the open field, light-dark box and trauma-reminder tests following protected predator exposure and chronic treatment with methylphenidate in male mice.**

| **Open Field** | | | |
| --- | --- | --- | --- |
| **Group** | **Number of entries** | **Time spent** | **Latency of first entry** |
| Non-stressed Met/Met, Vehicle | 75.20 ± 4.64 | 132.36 ± 10.67 | 5.71 ± 2.00 |
| Non-stressed Met/Met, Methylphenidate | 76.33 ± 4.89 | 103.14 ± 11.24 | 9.12 ± 2.12 |
| Stressed Met/Met, Vehicle | 71.02 ± 3.92 | 123.98 ± 9.01 | 4.23 ± 1.70 |
| Stressed Met/Met, Methylphenidate | 79.15 ± 4.07 | 99.14 ± 9.36 | 6.39 ± 1.76 |
| Non-stressed Val/Val, Vehicle | 69.75 ± 4.23 | 130.23 ± 9.74 | 4.00 ± 1.83 |
| Non-stressed Val/Val, Methylphenidate | 82.40 ± 4.64* | 105.82 ± 10.67 | 5.40 ± 2.01 |
| Stressed Val/Val, Vehicle | 61.70 ± 4.68 | 98.77 ± 10.71 | 9.48 ± 2.05 |
| Stressed Val/Val, Methylphenidate | 84.29 ± 3.92** | 110.50 ± 9.01 | 7.14 ± 1.79 |
| **Light-dark box** | | | |
| **Group** | **Number of entries** | **Time spent** | **Latency of first entry** |
| Non-stressed Met/Met, Vehicle | 41.00 ± 11.08 | 305.04 ± 34.89 | 9.91 ± 4.72 |
| Non-stressed Met/Met, Methylphenidate | 41.33 ± 11.68 | 237.20 ± 36.78 | 13.96 ± 4.97 |
| Stressed Met/Met, Vehicle | 33.07 ± 9.36 | 287.44 ± 29.49 | 21.92 ± 3.99 |
| Stressed Met/Met, Methylphenidate | 43.85 ± 9.72 | 257.26 ± 30.60 | 15.13 ± 4.14 |
| Non-stressed Val/Val, Vehicle | 32.58 ± 10.11 | 305.97 ± 31.85 | 9.70 ± 4.30 |
| Non-stressed Val/Val, Methylphenidate | 65.20 ± 11.08 | 210.03 ± 34.89 | 11.60 ± 4.72 |
| Stressed Val/Val, Vehicle | 34.00 ± 11.11 | 262.95 ± 33.87 | 19.32 ± 4.85 |
| Stressed Val/Val, Methylphenidate | 47.64 ± 9.36 | 302.11 ± 29.49 | 10.95 ± 3.99 |
| **Trauma-reminder test** | | | |
| **Group** | **Number of approaches** | **Time spent** | **Latency of first approach** |
| Non-stressed Met/Met, Vehicle | 34.90 ± 2.78 | 126.44 ± 16.92 | 2.14 ± 2.45 |
| Non-stressed Met/Met, Methylphenidate | 42.89 ± 2.93 | 110.20 ± 17.84 | 1.05 ± 2.58 |
| Stressed Met/Met, Vehicle | 30.17 ± 2.54 | 113.52 ± 15.45 | 9.29 ± 2.24 |
| Stressed Met/Met, Methylphenidate | 39.50 ± 2.64* | 142.99 ± 15.54 | 9.48 ± 2.55 |
| Non-stressed Val/Val, Vehicle | 31.50 ± 2.75 | 126.85 ± 15.78 | 5.03 ± 2.44 |
| Non-stressed Val/Val, Methylphenidate | 38.80 ± 2.79 | 110.01± 16.95 | 3.04 ± 2.45 |
| Stressed Val/Val, Vehicle | 27.70 ± 2.97 | 131.90 ± 16.98 | 5.94 ± 2.66 |
| Stressed Val/Val, Methylphenidate | 41.69 ± 2.44** | 128.18 ± 14.84 | 4.98 ± 2.15 |

Data are presented as mean ± SEM for the number of entries/approaches, time spent and latency of first entry/approach in the aversive area across all the behavioral tests. The aversive areas are the center area, the lit chamber and the odor tube in the open field, light-dark box and trauma-reminder tests, respectively. In the open field test, a three-way ANOVAs (genotype × stress × methylphenidate) revealed a main effect of methylphenidate (F_1,84_ = 12.87; *p* < 0.001; genotype × methylphenidate: F_1,84_ = 4.41; *p* < 0.05) on the number of entries in center area, with increased number of entries in the center area in Val/Val mice treated with methylphenidate vs. corresponding group treated with vehicle. Methylphenidate also decreased the time spent in the center area (F_1,84_ = 5.48; *p* < 0.05), and a genotype × stress interaction (F_1,84_ = 4.53; *p* < 0.05) was found on the latency of first entry in the center area. In the trauma-reminder test, methylphenidate increased the number of approaches to cat odor tube (F_1,84_ = 26.19; *p* < 0.001). **p* < 0.05 and ***p* < 0.01 vs. corresponding group treated with vehicle following Tukey’s *post hoc* test.
